# Recurrent paraganglioma of the vulva: A rare case report and review of the literature

**DOI:** 10.3389/fonc.2022.961666

**Published:** 2022-08-26

**Authors:** Wenzhi Kong, Qingxi Qu, Shiqian Zhang

**Affiliations:** Department of Obstetrics and Gynaecology, Qilu Hospital of Shandong University, Jinan, China

**Keywords:** valval paraganglioma, functional tumour, diagnosis, treatment, SDHB genes

## Abstract

**Purpose:**

Vulva paragangliomas are rare and usually misdiagnosed or missed, especially in juveniles. Our aim was to summarize the clinical characteristics and treatments of vulva paragangliomas.

**Methods and results:**

We present a case of a 17-year-old Chinese patient with functional paraganglioma from the vulva that was misdiagnosed as clear cell carcinoma. She had suffered from severe headaches, palpitations, sweating, pallor and hypertension. The vaginal wall was invaded by this mass. The tumour was surgically removed smoothly. However, the disease recurred 7 years after surgery, and the patient was treated again. Personalized genetic testing was performed while recovering, and the results suggested that the patient had a germline mutation in the Succinate Dehydrogenase subunit B (SDHB) gene. Now, the patient has been discharged successfully, her blood pressure has returned to normal and some of her clinical symptoms disappeared. A review of the literature concerning the topic is also presented, there have been only 2 cases of paraganglioma of the vulva and 11 cases of vaginal paraganglioma since 1955.

**Conclusion:**

Our case describes a recurrent vulvovaginal paraganglioma with SDHB gene mutation and the largest tumor diameter to date. The diagnosis and treatment process of this case can provide reference for the management of other similar patients.

## Introduction

Pheochromocytomas (PCCs) and paragangliomas (PGLs) (together referred to as PPGLs) are endocrine tumours originating from neural crest–derived cells of the adrenal medulla or from the sympathetic or parasympathetic paraganglia. Traditionally considered a “one in a million” disease, PPGLs have shown a rising incidence during the last 40 years, from 1.4 per million person-years in 1977 to 6.6 in 2015, constituting a 4.8-fold increase (1). PPGLs have the highest reported degree of heritability among all tumors. When currently known germline mutations are taken into account, around 30% to 40% of patients with PPGLs are affected by germline mutations in various susceptibility genes, and a further 35% to 40% show somatic driver mutations (2), and 50%-70% of childhood PPGL is associated with germline mutations. More than 20 germline mutations are known to be associated with PPGLs (3), Krebs cycle–related PPGLs are currently regarded as the most aggressive paraganglionic tumours, with germline Succinate Dehydrogenase subunit B (SDHB) mutations being among the strongest genetic risk factor for in development of metastatic PPGLs. The therapy of choice is surgery whenever possible; for inoperable disease, systemic therapy options include chemotherapy, radionuclide therapy, and tyrosine kinase inhibitors. PPGLs are mostly distributed in the adrenal glands, neck, mediastinum, and retroperitoneum, but they rarely occur in the vaginal wall and vulva. They are even rarer in juveniles. According to previous reports, there have been only 2 cases of PGLs of the vulva and 11 cases of vaginal PGLs since 1955 (4–16) (**Table 1**). Herein, we reported a vulval functional recurrent PGL.

**Table 1:** The summarized table of all vulva and vaginal paraganglioma cases reported.

author	Year	Age	location	size	functional	symptoms	gene mutation	Recurrene during follow-up
Plate, W. P (6).	1955	66	vaginal	walnut size	nonfunctional	vaginal hemorrhage	Not run	no
Pezeshkpour G (7).	1981	22	vaginal	3*2.5*1.5 cm	nonfunctional	asymptomatic	Not run	no
Colgan,T.J (4).	1991	58	vulva	1cm	nonfunctional	vulua pain and tenderness	Not run	no
Parkes, S. E (8).	1998	11	vaginal	5cm	nonfunctional	vaginal bleeding	Not run	no
Hassan, A (9).	2003	24	vaginal	2.5 cm	functional	hypertension, tachycardia and heart failure	Not run	no
Brustmann, H (10).	2007	33	vaginal	1.9 and 1.4 cm	nonfunctional	vaginal bleeding	Not run	no
Shen, J. G (11).	2008	38	vaginal	3.0 cm	functional	Paroxysmal headaches, chest distress, palpitation	Not run	no
Akl, M. N (12).	2010	65	vaginal	2.5*2.3*2cm	nonfunctional	asymptomatic	Not run	no
Liu,Y (5).	2013		vulva	3.2*2.3*1.5 cm	nonfunctional	asymptomatic	Not run	no
Cai, T (13).	2014	17	vaginal	3.5*3.0*2.5 cm	functional	vaginal bleeding	Not run	no
Sharma, S (14).	2018	28	vaginal	3*3 cm	nonfunctional	asymptomatic	Not run	no
Wong, R. W (15).	2020	15	vaginal	3 cm	nonfunctional	Irregular heavy menses, dysmenorrha, and anemia	Finding genetic mutations	no
Wang, Z (16).	2021	44	vaginal	3.5 cm	functional	hypertension, palpitations and dizziness	No mutations were found	Under follow up
Kong W.	2022		vulva	6*4*4cm	functional	hypertension,headaches, palpitations,	Finding genetic mutations	Recurrence 7 years later

## Case presentation

**Figure 1:**
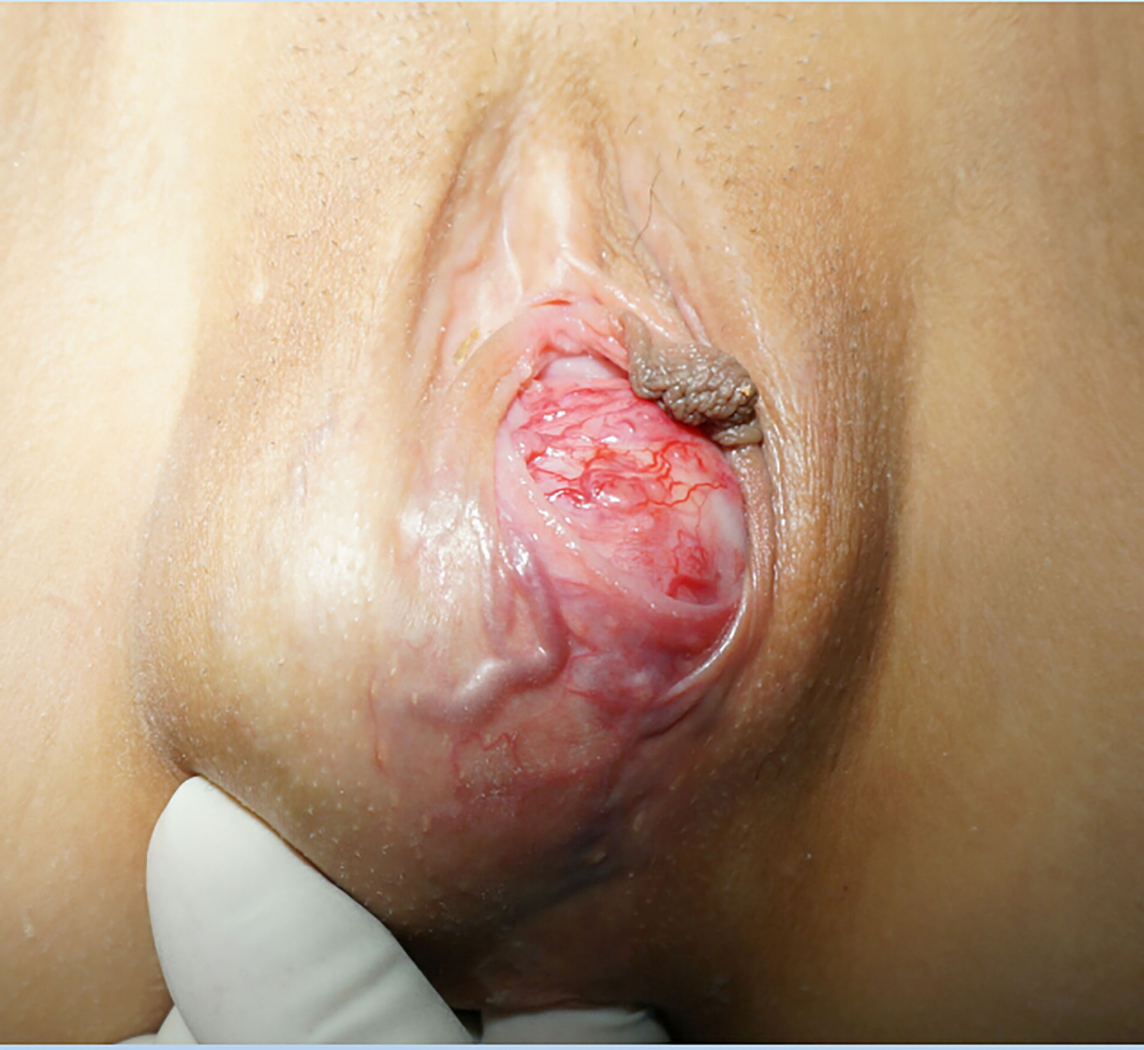
The tumour was found to be located below the right labia majora, and its surface was covered with massive, engorged blood vessels.

A 17-year-old girl who had regular menstrual periods and no history of marriage or sexual activity was referred to the hospital for a hard lump located on the right side of the vulva for approximately 2 years. The lump progressively increased in size, with mild pain after mild physical activity. She had suffered from severe headaches, palpitations, sweating, and pallor after intense physical activity, such as sports and physical labor. These symptoms were especially common after activities involving local compression (e.g. cycling and defecation). Upon gynaecologic examination, an immobile, solid, and involved mass with no bleeding was found on the inferior margin of the right labia majora and the lateral margin of the hymen (**Figure 1**). Digital rectal examination (DRE) showed this mass to be closely associated with the vaginal wall and rectal wall. At the same time, inpatient evaluation revealed a blood pressure of 140–170/100–120 mmHg and a pulse rate of 90–120 beats/minute. These issues were not relieved by either metoprolol prolonged-release tablets or nifedipine tablets.

**Figure 2:**
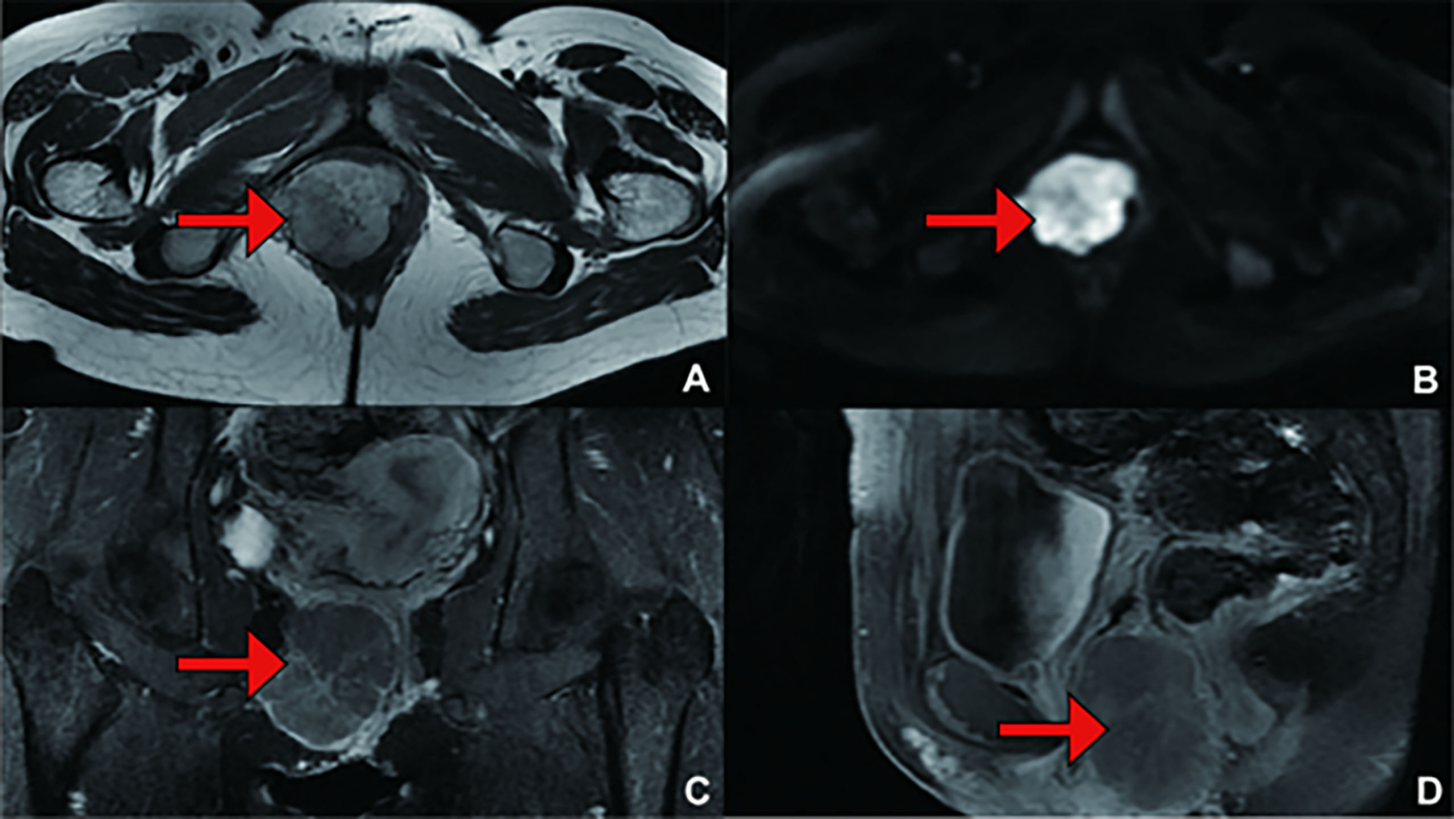
(Primary Treatment) Magnetic resonance imaging (MRI) scans: A mass on the right vulva measuring 6.0 cm*5.5 cm*4.3 cm in size, involving the right levator ani muscle, was observed. The tumour invaded the vaginal wall and disrupted the continuity of its mucous. **(A)** T1 weighted imaging (T1WI); **(B)** Diffusion Weighted Imaging (DWI); **(C)** Enhanced; **(D)** Enhanced.

A magnetic resonance imaging (MRI) scan of the pelvis with intravenous contrast revealed a mass on the right vulva measuring 6.0 cm*5.5 cm*4.3 cm in size, involving the right levator ani muscle. In addition, the tumour invaded the vaginal wall and disrupted the continuity of its mucous (**Figure 2**). Computed tomography (CT) scanning of the abdominal and female tumour markers were normal, while plasma and catecholamine concentrations were measured and found to be significantly higher than normal (**Table 2**). Fine needle aspiration cytology (FNAC) of the mass performed in other centres revealed clear cell carcinoma, but our centre denied this conclusion. To make a clear diagnosis and determine a suitable care plan, the patient underwent core needle biopsy (CNB) again in our centre. The biopsy tissue was sent for pathological and immunohistochemical examination, and the diagnosis was confirmed to be PGLs, synaptophysin (Syn)+, chromogranin A (CgA)+, S100+, Desmin+, HMB45 -, MelanA -, cyto-keratin (CK)-, CD10 -, CD68 -, CD34 -, CD31 -, PAX-8 -.

**Table 2:** Plasma catecholamine concentrations (primary treatment).

Item	Result	Unit	Hint	Reference Value
**E**	40.23	Pg/mL		0.00–100.00
**NE**	3747.16	Pg/mL	↑	0.00–600.00
**DA**	167.85	Pg/mL	↑	0.00–100.00

E, epinephrine; NE, norepinephrine; DA, dopamine. ↑ Indicates that the result is higher than the normal reference range.

After oral administration of carvedilol and phenoxybenzamine for 14 days and intravenous fluids for 5 days, the effective circulating blood volume improved. Tumour resection was performed under general anaesthesia. During the operation, the tumour was found to be located below the right labia majora, and its surface was covered with massive, engorged blood vessels (**Figure 1**). The right posterior lateral vaginal mucosa was involved. At the moment that the surgeon touched the tumour during the operation, the patient’s arterial blood pressure and heart rate increased dramatically to 203/132 mmHg and 114 beats/min, respectively. However, she suffered from severe hypotension (40/30 mmHg) after complete tumour excision. This was managed by blood transfusion and vasopressor administration until her vital signs became stable. After surgery, blood pressure and heart rate were 100–110/60–70 mmHg and 90–100 beats/min, respectively, and the patient showed no manifestations suggestive of catecholamine release.

**Figure 3:**
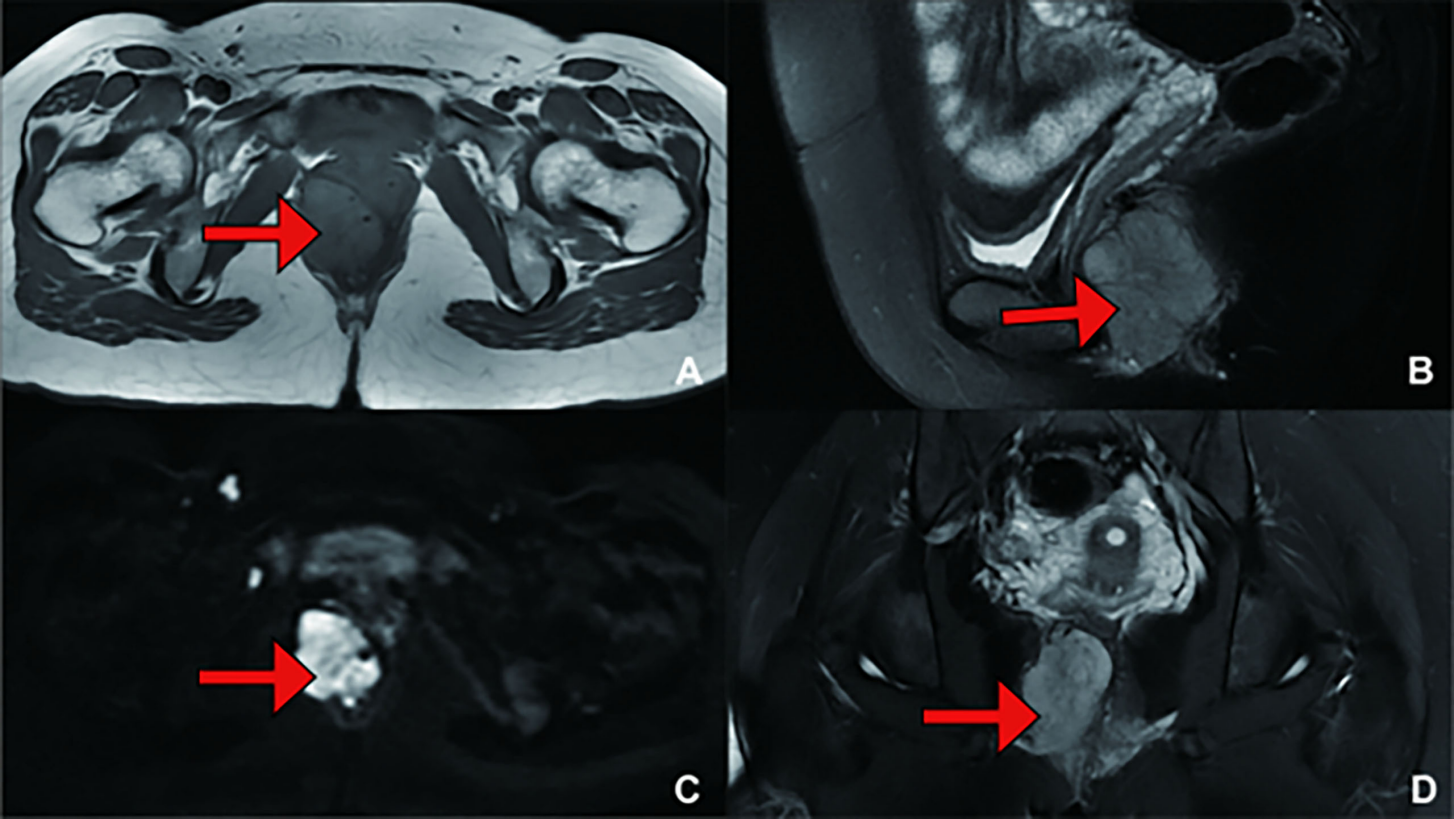
(Recurrence Treatment) Pelvic MRI showing a mass measuring 4.3 cm*3.6 cm*5.4 cm in size, suggesting that the lesion was closely related to the right posterior vaginal wall and anterior rectal wall. **(A)** T1 weighted imaging (T1WI); **(B)** T2 weighted imaging (T2WI); **(C)** Diffusion Weighted Imaging (DWI); **(D)** T2 weighted imaging (T2WI).

**Figure 4:**
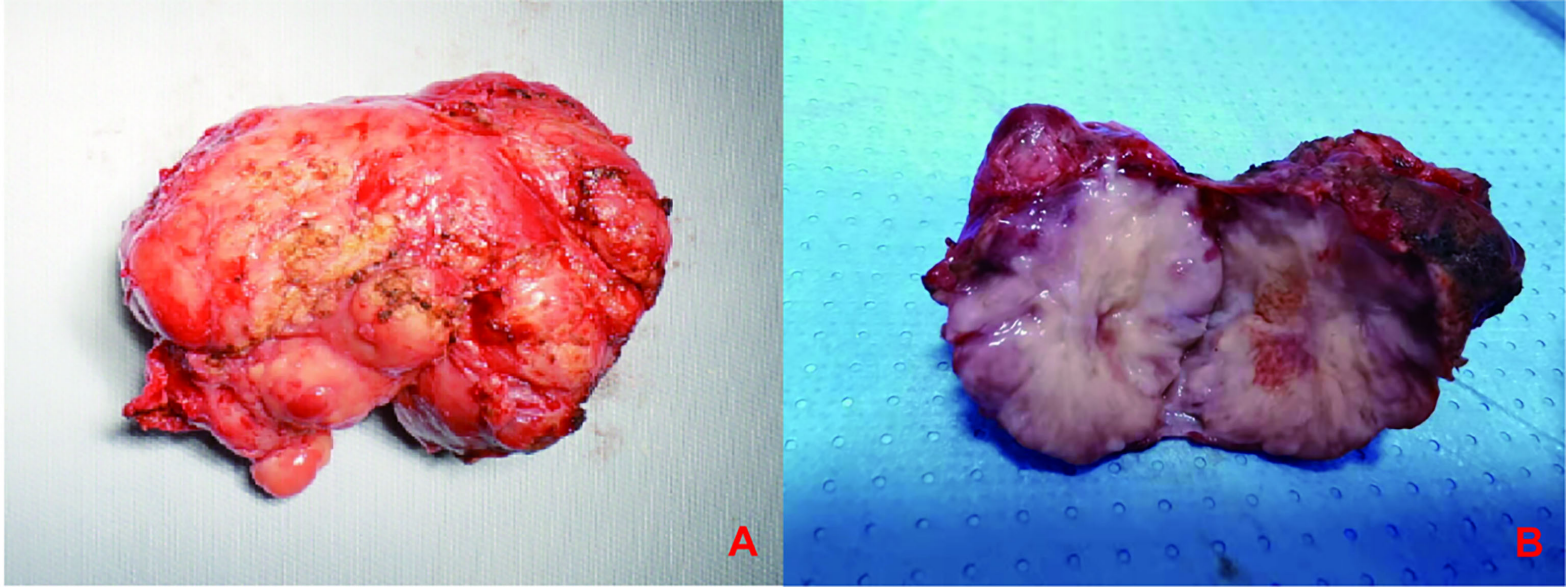
**(A)** The mass was dissected under the table and was approximately 6*4*4 cm in size, **(B)**The mass was hard and have a greyish white myxoma-like cut surface.

The patient did not have any active complaints in the following 2-year period. In a routine examination 7 years later, a mass was detected again at the same place, and the patient’s blood pressure was increased. In the interval between the initial onset and recurrence, the patient completed childbirth. The levels of plasma catecholamines and their metabolite were examined to determine disease recurrence (**Table 3**). Whole-body CT examination was performed to exclude primary tumours in other sites, and pelvic scan plus enhanced MRI (**Figure 3**) and transanal ultrasound were used to clarify the extent of tumour invasion. The patient underwent surgery again. At this time, the resected mass was approximately 6*4*4 cm in size, hard and have a greyish white myxoma-like cut surface (**Figure 4**).

**Table 3:** Plasma catecholamine concentrations (recurrence treatment).

Item	Result	Unit	Hint	Reference Value
**NE**	35.00	nmol/L	↑	0-5.17
**E**	0.14	nmol/L		0-0.34
**NMN**	14.40	nmol/L	↑	0-0.71
**MN**	0.14	nmol/L		0-0.42
**DA**	<0.14	nmol/L		0-0.31
**HVA**	69.40	nmol/L		14.27-163
**VMA**	243.00	nmol/L	↑	0-62

NE, norepinephrine; E, epinephrine; DA, dopamine; NMN, normetanephrine; MN, metanephrine; DA, dopamine; HVA, homovanillic acid; VMA,vanillylmandelic acid. ↑ indicates that the result is higher than the normal reference range.

**Figure 5:**
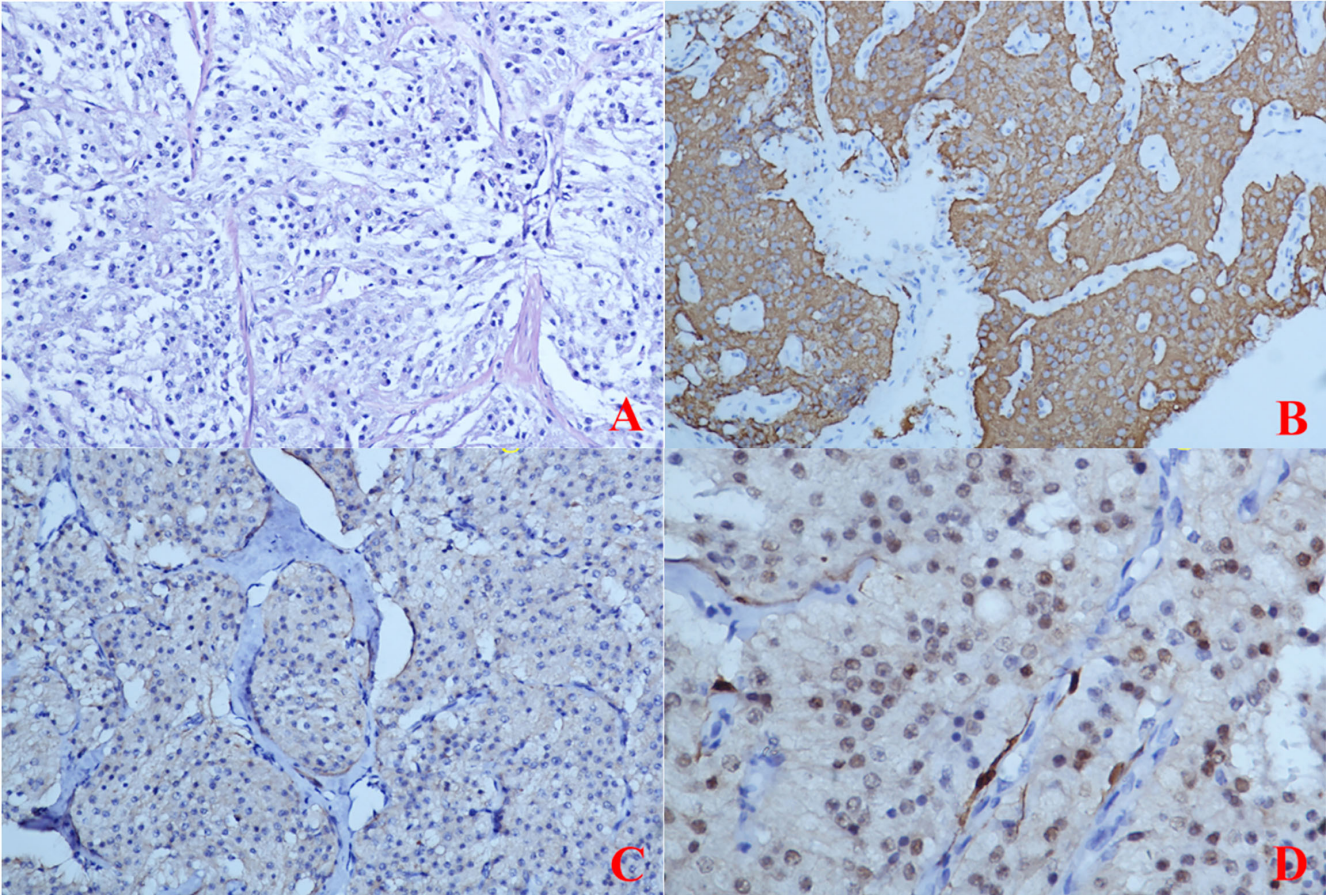
(Primary Treatment) Immunohistochemical staining: **(A)** The tumour cells were composed of chief cells and sustentacular cells, and they were clustered in small nests, here called zellballen, demarcated by delicate fibrous stroma and capillaries. **(B)** Immunohistochemical staining CgA(+); **(C)** Immunohistochemical staining SYN(+); **(D)** Immunohistochemical staining S-100(-).

**Figure 6:**
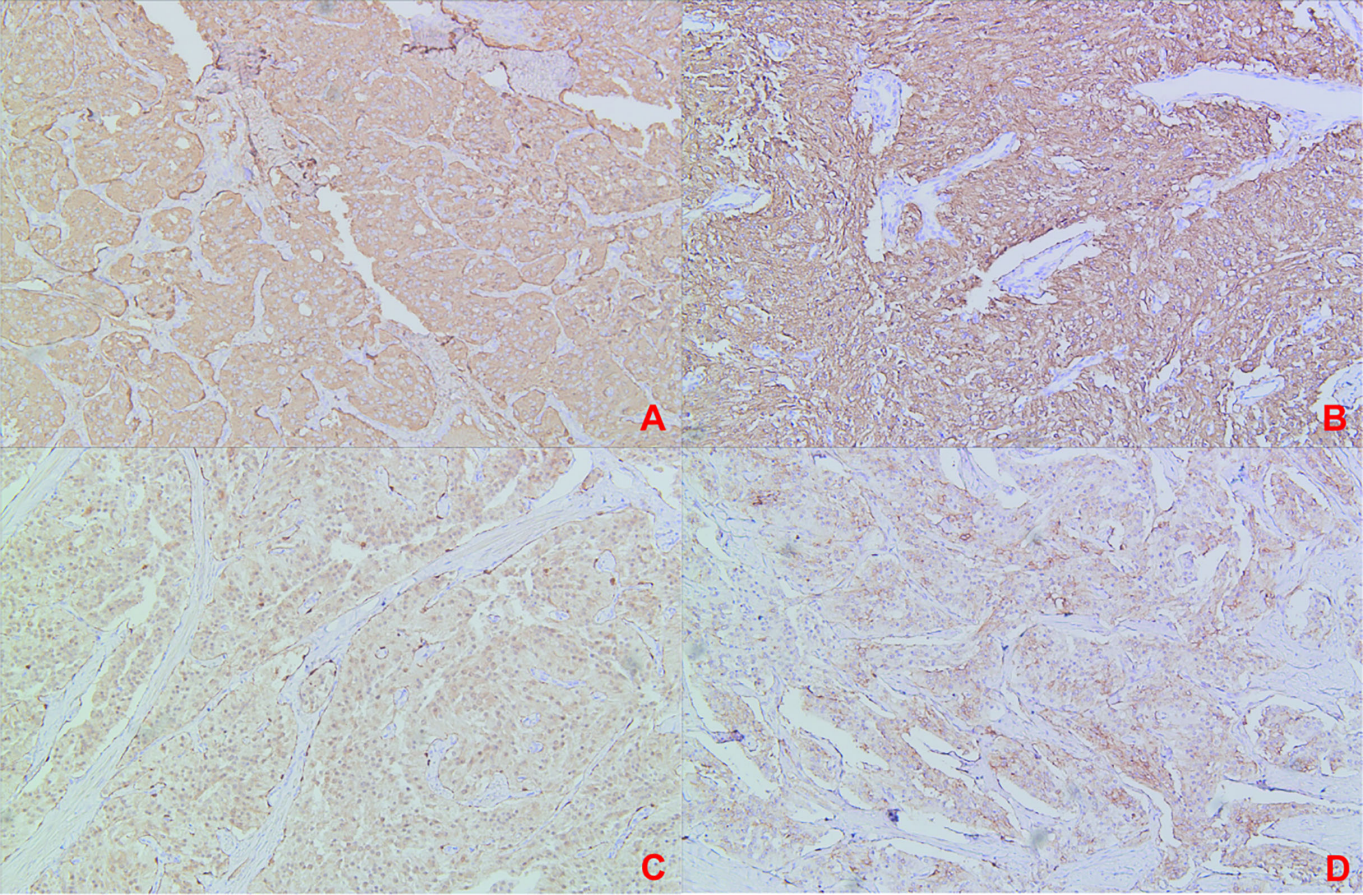
(Recurrence Treatment)Immunohistochemical staining: **(A)** Immunohistochemical staining CgA(+); **(B)** Immunohistochemical staining SYN(+); **(C)** Immunohistochemical staining S-100(+); **(D)** Immunohistochemical staining SSTR2 (+).

Comparing the pathology of the two excised tissues, the tumour cells were composed of chief cells and sustentacular cells, and they were clustered in small nests, here called zellballen, demarcated by delicate fibrous stroma and capillaries. The uninvolved margin around the neoplasm was at least 5 mm. The immunohistochemistry results at the time of primary treatment were as follows: the tumour was found to be positive for Syn, CgA, and Desmin but negative for protein S-100, human melanoma-associated antigen, MelanA, CK, CD10, CD68, CD31, and PAX-8 (**Figure 5**). The immunohistochemistry results at the time of recurrence treatment were as follows: CgA (+), CD56 (+), Syn (+), somatostatin receptor (SSTR2) (+), S-100 (+), SOX-10 (individual cells +), CK (-), and P53 (-), with a Ki-67 positive rate of approximately 2-3% (**Figure 6**). According to the Grading System for Adrenal Pheochromocytoma and Paraganglioma (GAPP) score which published in 2014 by Dr Kimura and co-workers (3), the patient can be rated 6 points, including 1 point for large and irregular cell nests, 2 points for high cellularity (> 250 cells/U), 1 point for capsular or vascular invasion, 1 point for Ki-67 labelling index 2-3%, and 1 point for catecholamine-type was NE.

After recurrence treatment, personalized genetic testing was performed during recovery, and the results suggested that the patient had a germline mutation in the SDHB gene. Assessment by Medical Genetics identifed a germline SDHB exon 7 splice site mutation (NM_003000.3(SDHB):c.765+1G>A). The minor allale frequency is 51%. According to American College of Medical Genetics and Genomics (ACMG) guidelines (17), the variation was preliminarily determined as pathogenic variation PVSI+PS4+PM2. The germline SDHB exon 7 splice site mutation described in the case has not been reported previously, which was predicted to disrupt splicing and lead to an altered and likely non-functional protein product. On this basis, genetic testing was performed for her immediate family. The genetic test results of the patient’s son were normal without phenotype and did not carry the same pathogenic mutation as the patient, suggesting that the patient’s son is less likely to develop the disease. The genetic test results of the patient’s mother were normal as well. As for the patient’s father, he did not perform the genetic test because of some personal reasons. Since the mode of inheritance is autosomal dominant, if the patient has another child, there is a 50% chance of the offspring having the disease, and prenatal counselling is recommended for another child.

## Discussion

PPGLs are rarely reported along the genital tract and are even rarer in juveniles. Only three cases of juvenile vaginal PGLs have been reported, with the patients’ aged 11, 17 and 15 years (8, 13, 15). The latter involved functional tumours (13). The largest diameter of any previously reported PGL of the vagina or vulva was 5 cm, making the current case the largest functional PGL of the vulva ever reported by diameter (8). Only 2 genetic investigations were documented in the reported cases (15, 16).

The clinical manifestations of PPGLs are associated with unregulated secretion of catecholamines and with the location of the tumour. Excessive secretion of catecholamines by functional tumours is largely responsible for paroxysmal or persistent hypertension and symptoms such as palpitations, headaches, and hyperhidrosis. It can even lead to lethal cardiovascular complications, including shock and hypertensive crisis, which classically characterize these tumours (18). There have been only 4 cases of functional tumours among vaginal and vulval PGLs reported since 1955 (9, 11, 13, 16). The 2 reported patients with vaginal PGLs suffered from a cascade of events, including acute pulmonary oedema, hypertensive crisis, severe headaches, and other symptoms, after an attempted biopsy or excision of the mass (9, 13). Another functional case reported a 16-year history of intermittent strong paroxysmal headaches, palpitations, and chest distress. A similar episode took place during the operation that was performed to remove her tumour (11). The other functional case reported a 6-year history of paroxysmal hypertension, dizziness and palpitations (16). For these reasons, any manipulation of the tumour must be gentle, as brief as possible. This may prevent the release of catecholamines. Some cases presented with abnormal vaginal bleeding (6, 8, 10, 13).

The diagnosis of PPGLs includes qualitative and localization diagnosis. The biochemical presentation of excessive catecholamine production is an essential step in the diagnosis of functional PPGLs (19). Plasma or urine concentrations of metanephrine (MN) and normetanephrine (NMN) are preferred for biochemical examination. MNs are intermediate metabolites of catecholamines. Unlike norepinephrine (NE) and epinephrine (E), they are continuously produced in PPGL tumours, are not easily degraded by hydrolytic enzymes after secretion and can be stably present in the blood (20, 21). For SDHB genes mutations like this patient that result of stabilization of hypoxia-inducible factors and epigenetic silencing of phenylethanolamine N-methyltransferase, the enzyme that converts NE into E, the focus should be on NMN since these tumours do not produce E (22). CT scanning and MRI are traditional positioning measurements. MRI is the preferred procedure for paediatric patients. The specificity of ¹²³I-metaiodobenzylguanidine (MIBG) scanning can be as high as 95–100%. In a report of vaginal PGL published by Zhan Wang (16), 131I-MIBG examinations revealed an obvious contractive accumulation in the perineum area. Scintigraphy of tumour somatostatin receptor (SSTR) and positron emission tomography (PET) are useful in the diagnosis of multifocal, metastatic disease and occult PGLs (19). In a report of vaginal PGL published by Tao Cai (13), two PGLs were found by PET: one was located in the vagina, and the other was located in the pelvic area.

The pathological diagnosis of PPGLs is also the gold standard for other tumours. Complete pathological examination can help distinguish PPGLs from other tumours, such as rhabdomyosarcoma, haemangioma, and leiomyoma. The diagnosis of PPGL is usually performed using histology plus immunohistochemical detection of CgA+, Syn+, GATA3+, S100+, tyrosine hydroxylase (TH)+, dopamine beta-hydroxylase (DBH)+ while being negative for CK (23). In addition to the biomarkers that confirm the diagnosis of PPGLs discussed above, there are important biomarkers that relate to the genetic background of PPGLs, that may guide genetic testing. For example, The 2022 World Health Organization(WHO) classification encourages routine use of SDHB immunohistochemistry since loss of SDHB expression is a surrogate marker in SDHx (x standing for any of the SDH genes) related pathogenesis (24). PPGLs with immunohistochemical loss of SDHB expression are classified as SDH-deficient PPGLs. This tumour is easily misdiagnosed by FNAC and intraoperative frozen sectioning. Dr. Sheila reported a vaginal PGL in an 11-year-old girl. She was misdiagnosed with rhabdomyosarcoma by frozen sectioning (8). Our patient was misdiagnosed with clear cell carcinoma by FNAC. Therefore, the pathological diagnosis of PGL should be completed by an experienced pathologist.

PPGLs does not respond well to chemotherapy or radiation, so complete surgical resection remains the standard of care (25). Sufficient preoperative preparation, including oral alpha-adrenoceptor blockade and intravenous fluids to increase blood volume, is very important. Excessive catecholamines from the tumour can lead to vasoconstriction of patients and cause a sharp rise in blood pressure. Therefore, it is necessary to expand the capacity periodically before operation to dilate the blood vessels and maintain the blood pressure of patients in a certain range, so as to prevent PGLs from being touched during operation, resulting in a rise in blood pressure of patients and resulting in dangerous surgery. The recommendations on preoperative preparation with alpha-adrenoceptor blockade are based on optimal care of patients, both before and during the surgery when a cardiovascular emergency and crisis may occur. Peri-operative mortality is as high as 30% in the absence of sufficient preoperative preparation. This is due to indefinite diagnosis or misdiagnosis. This rate may drop to less than 3% if there is adequate preoperative preparation (26). In addition, the blood supply associated with PGL is extremely rich, Mohamed N. Akl MD successfully used an intervention of embolization for the uterine artery to reduce bleeding (12).

PPGLs are rare tumours and at least 30% are part of hereditary syndromes (27). PPGLs have the highest reported degree of heritability among all tumors. More than 20 germline mutations are known to be associated with PPGLs (3), about half of patients with metastatic disease harbour PPGLs susceptibility genes mutations. Germline mutations are most commonly detected in the SDHB, REarranged during Transfection (RET), von Hippel-Lindau(VHL) and NeuroFibromatosis type 1(NF1) genes. SDHB gene mutation are the most common SDHx mutations. The SDHB gene mutation is autosomal dominant and is localized at 1q36-q35 with eight exons, encoding a tricarboxylic acid cycle regulator of the mitochondrial complex II (27). The impairment of genes of the Krebs cycle leads to the accumulation of the oncometabolites succinate, fumarate, or 2-hydroxyglutarate. This in turn promotes DNA hypermethylation, inactivation of tumor suppressor genes, resulting in less hypoxia-inducible factor (HIF)-α hydroxylation and significantly lower HIF-α ubiquitination/degradation. This causes HIF-α stabilization, mitochondrial DNA impairment, collagen instability, and most likely an abnormal immune microenvironment. Only 2 genetic investigations were documented in the reported cases (15, 16). Wong, R. W (15).reported a vaginal PGL in an 15-year-old girl with a heterozygous deletion of exon 1 of the SDHB gene. Wang, Z (16). investigated 36 most common mutated genes related to PPGL through target sequencing, however, the results turned out to be negative.

Although all PPGLs and all genotypes have a potential for developing metastatic disease, mutations in the SDHB gene are associated with the highest risk of metastatic disease (30–70%). Therefore, SDHB mutations suggest that patients have a poor prognosis and should be closely monitored (28, 29). An appropriate follow-up program should be selected according to the genotype of PGL patient. Patients with SDHB gene mutations should be examined annually for blood pressure and biochemistry and every 2 years for whole-body MRI (30). A multicentric retrospective study indicated that early knowledge of genetic status had a positive impact on the management and clinical outcome of patients with a germline SDHx or VHL mutations (31). Mutation testing for the SDHB oncogene in all patients clinically diagnosed with PPGLs is beneficial not only for the confirmation of diagnosis and prognosis assessment of PPGLs patients but also for the early diagnosis and early treatment of patients’ family members.

## Data availability statement

The original contributions presented in the study are included in the article/supplementary material. Further inquiries can be directed to the corresponding author.

## Ethics statement

The studies involving human participants were reviewed and approved by the Ethics Committee of Qilu Hospital of Shandong University KYLL-202203-018. Written informed consent was obtained from the relevant individual and minors' legal guardian, for the publication of any potentially identifiable images or data included in this article.

## Author contributions

SZ screened the study subjects, participated in discussions and provided expertise and feedback. QQ assessed the patients’ pathologic complications and participated in the manuscript revision. WK wrote the clinical information and manuscript. All authors contributed to the article and approved the submitted version.

## Funding

This work was supported by the Natural Foundation of Shandong Province (No. ZR2021QH011).

## Conflict of interest

The authors declare that the research was conducted in the absence of any commercial or financial relationships that could be construed as a potential conflict of interest.

## Publisher’s note

All claims expressed in this article are solely those of the authors and do not necessarily represent those of their affiliated organizations, or those of the publisher, the editors and the reviewers. Any product that may be evaluated in this article, or claim that may be made by its manufacturer, is not guaranteed or endorsed by the publisher.
